# Case-mix and the use of control charts in monitoring mortality rates after coronary artery bypass

**DOI:** 10.1186/1472-6963-7-63

**Published:** 2007-04-30

**Authors:** Tom Marshall, Mohammed A Mohammed

**Affiliations:** 1 Department of Public Health & Epidemiology, University of Birmingham, Birmingham, B15 2TT, UK

## Abstract

**Background:**

There is debate about the role of crude mortality rates and case-mix adjusted mortality rates in monitoring the outcomes of treatment. In the context of quality improvement a key purpose of monitoring is to identify special cause variation as this type of variation should be investigated to identify possible causes. This paper investigates agreement between the identification of special cause variation in risk adjusted and observed hospital specific mortality rates after coronary artery bypass grafting in New York hospitals.

**Methods:**

Coronary artery bypass grafting mortality rates between 1994 and 2003 were obtained from the New York State Department of Health's cardiovascular reports for 41 hospitals. Cross-sectional control charts of crude (observed) and risk adjusted mortality rates were produced for each year. Special cause variation was defined as a data point beyond the 99.9% probability limits: hospitals showing special cause variation were identified for each year. Longitudinal control charts of crude (observed) and risk adjusted mortality rates were produced for each hospital with data for all ten years (n = 27). Special cause variation was defined as a data point beyond 99.9% probability limits, two out of three consecutive data points beyond 95% probability limits (two standard deviations from the mean) or a run of five consecutive points on one side of the mean. Years showing special cause variation in mortality were identified for each hospital. Cohen's Kappa was calculated for agreement between special causes identified in crude and risk-adjusted control charts.

**Results:**

In cross sectional analysis the Cohen's Kappa was 0.54 (95% confidence interval: 0.28 to 0.78), indicating moderate agreement between the crude and risk-adjusted control charts with sensitivity 0.4 (95% confidence interval 0.17–0.69) and specificity 0.98 (95% confidence interval: 0.95–0.99). In longitudinal analysis, the Cohen's Kappa was 0.61 (95% confidence interval: 0.39 to 0.83) indicating good agreement between the tests with sensitivity 0.63 (95% confidence interval: 0.39–0.82) and specificity 0.98 (95% confidence interval: 0.96 to 0.99).

**Conclusion:**

There is moderate-good agreement between signals of special cause variation between observed and risk-adjusted mortality. Analysis of observed hospital specific CABG mortality over time and with other hospitals appears to be useful in identifying special causes of variation. Case-mix adjustment may not be essential for longitudinal monitoring of outcomes using control charts.

## Background

Monitoring hospital mortality is an integral part of the quality assurance process in health care provision [1,2]. Mortality rates are often made public and may therefore be used to rank healthcare providers [3-5]. The ranking process has been criticised for failing to take sufficient account of case-mix and failing to distinguish between variation due to chance and variation due to special causes [6]. It is generally recommended that mortality rates be adjusted for measurable case-mix factors [7,8] However a number of commentators have observed that adjustment is not without difficulty [9,10]. Others have debated the advantages of one method of case-mix adjustment over another [11,12]. There is also debate about whether clinical or administrative data are sufficient for case-mix adjustment [13,14].

Coefficients used for case-mix adjustment are often derived on the assumption that there are linear relationships between measurable case-mix factors such as age, sex, or co-morbidities and outcomes such as mortality. If the relationships are non-linear, or there are complex interactions between case-mix factors, or if the relationships change with time, case-mix adjustment may be misleading. Even if there are simple linear relationships between case-mix factors and mortality, there may also be systematic associations between case-mix factors and provider. This would result in confounding, in turn leading to over or under-adjustment. The issue of un-measurable case-mix factors presents further challenges to the case-mix adjustment methodology [15].

Given the difficulties in using adjusted mortality rates it is worth asking whether analysis of crude mortality rates has any useful role in delivering quality improvement. Where monitoring is intended to drive improvement, its principal aim should be to distinguish between common cause (chance) variation and special cause variation. Special cause variation is a signal to guide further investigation and learn from the results [16,17]. Special cause variation is identified using charting methods, such as variable life adjusted displays, cumulative sum charts or Shewhart control charts [18-20]. Monitoring with the aim of quality improvement is therefore best undertaken using charts.

The New York State Department of Health's cardiovascular reports provide a useful data source for exploring our research question [21]. These reports show observed mortality for all 41 cardiac surgery centres in New York State during 1994–2003. Using multivariate logistic regression, each report derives a predicted probability of mortality for each patient based on measured characteristics such as age, gender and co-morbidities. Each hospital therefore has an expected mortality rate: the sum of the predicted mortalities for its patients. The risk-adjusted mortality is a comparison of observed to expected numbers of death as a ratio with confidence intervals to reflect errors to due random sampling. Finally the reports also cite a risk adjusted mortality rate: this is the mortality rate that would be expected in the hospital had its patients been identical to the state wide mix [21].

A previous simulation study has confirmed that the risk adjustment methodology in these reports introduced significant bias into the calculation of risk adjusted mortality rates. Nevertheless, varying the case-mix did not appear to lead to the identification of different statistical outliers [22]. However this study only looked at outliers in a cross-sectional analysis of 1996 mortality data. It also defined outliers as hospitals whose 95% confidence intervals did not cross a standardized mortality ratio of one.

Shewhart control charts are intended to distinguish between variation that is consistent with a stable process (common cause variation) and variation that is not consistent with a stable process (special cause variation). They are intended to be used as part of a process of quality improvement. A process that shows common cause variation will continue to produce the same results. Improvement requires action to change the whole process. Special cause variation requires investigation to identify the special causes that are affecting the process. Special causes indicate which factors likely to be the most important influences on the process. This indicates which factors should be altered to effect improvement. Shewhart control charts consist of a central line (the mean) and two lines on either side of this central line (upper and lower control limits). Data points outside of the control limits or certain patterns in the data indicate special cause variation.

This paper investigates the effects of risk adjustment on the identification of special cause variation in hospital specific mortality rates after coronary artery bypass grafting in the New York State Department of Health's cardiovascular reports.

## Methods

Data were obtained from the New York State Department of Health's cardiovascular reports for the years from 1994 to 2003.

For each year's data cross-sectional P-charts were constructed for observed mortality rates and risk-adjusted mortality rates [23]. Control limits, which were calculated using an exact method based on the binomial distribution, were set at 99.9% probability (three standard deviations from the mean), and hospitals with mortality rates outside of the control limits were identified as indicating special cause variation. This kind of analysis identifies whether in any specific year, any individual hospital's mortality rate shows special cause variation in relation to other New York hospitals. Cohen's Kappa statistic was calculated for agreement between special cause variation in observed mortality rate and special cause variation in risk adjusted mortality rate [24]. In addition, test characteristics (sensitivity and specificity) were calculated, using special cause variation in risk-adjusted mortality rate as the reference standard.

Longitudinal P-charts were constructed for observed mortality rates and risk-adjusted mortality rates within each hospital over time. The average mortality rate for the hospital over ten years was calculated from the average of all ten years' data. Exact 99.9% probability control limits based on the binomial distribution were calculated for each year from the numbers of cases and numbers of deaths within the hospital. Special cause variation was pre-defined as: a single data point outside the 99.9% probability limits; two out of three successive data points outside the 95% probability limits (two standard deviations from the mean); five or more successive data points on one side of the mean (which have probability of ≤ 0.03 of occurring by chance)[25]. Hospitals with incomplete data (n = 15) were excluded. Longitudinal analysis of this kind identifies whether any individual hospital's mortality rate shows special cause variation from its own long-term mortality rate. The Cohen's Kappa statistic was calculated for agreement between special cause variation in observed and risk adjusted mortality rates. Test characteristics were also calculated, regarding special cause variation in risk-adjusted mortality as the reference standard.

Longitudinal P-charts were produced for expected mortality rates in each hospital. Expected mortality rates are a summary measure of patient case-mix profiles. Longitudinal analysis of this kind identifies whether any individual hospital's expected mortality rate (and hence its patients' case-mix profile) shows special cause variation in relation to its own long-term expected mortality rate. Longitudinal run charts were also produced for the total number of cases in each hospital.

## Results

### Cross-sectional analysis

**Figure 1:**
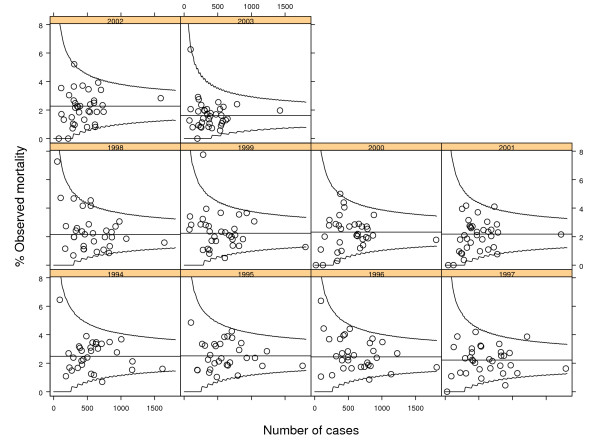
Cross-sectional P-charts of observed (crude) mortality after coronary artery bypass grafting in New York hospitals 1994–2003 for all hospitals. Horizontal line is the mean mortality with exact upper and lower control limits shown as (smoothed) solid curves.

**Figure 2:**
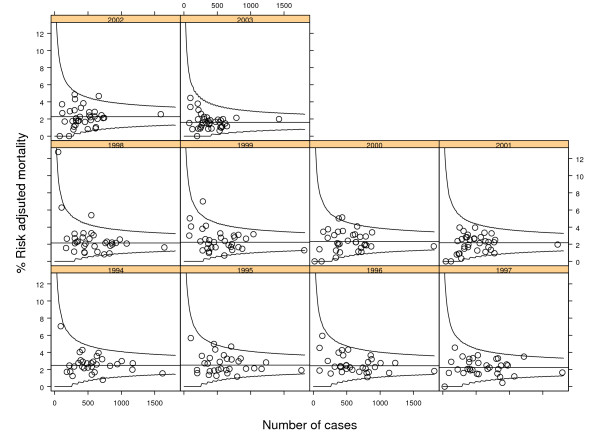
Cross-sectional P-charts of risk-adjusted mortality after coronary artery bypass grafting in New York hospitals 1994–2003 for all hospitals. Horizontal line is the mean mortality with exact upper and lower control limits shown as (smoothed) solid curves.

Figure 1 and Figure 2 show the cross-sectional control charts for crude and risk-adjusted annual mortality respectively. In the crude cross-sectional charts 11 signals of special cause variation (6 low and 5 high) were noted compared with 10 signals of special cause variation (2 low and 8 high) from the risk-adjusted charts. Table 1 compares the number of special cause signals from Figure 1 and Figure 2 as a two-by-two table. The associated Cohen's Kappa statistic is 0.54 (95% confidence interval 0.28–0.78) indicating moderate agreement.

**Table 1:** Cross-sectional analysis: special cause variation in Observed and Risk-Adjusted mortality rates for coronary artery bypass grafting surgery 1994 to 2003 in New York hospitals.

Observed mortality rate	Risk Adjusted mortality rate			
	
	Special cause variation (low)	Common cause variation	Special cause variation (high)	Total
Special cause variation (low)	2	4	0	6
Common cause variation	0	281	6	287
Special cause variation (high)	0	3	2	5

Total	2	288	8	298

If special cause variation on risk-adjusted mortality is taken to be the reference standard, the analysis of observed mortality has a sensitivity of 0.40 (95% confidence interval: 0.17 to 0.69) as a test for special cause variation and a specificity of 0.98 (95% confidence interval: 0.95 to 0.99).

### Longitudinal analysis

**Figure 3:**
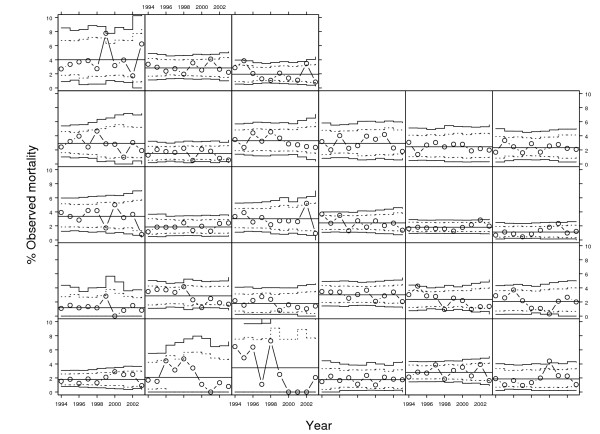
Longitudinal P-charts of observed mortality after coronary artery bypass grafting in New York hospitals 1994–2003 for 27 hospitals (each panel). Horizontal line is the mean mortality with exact upper and lower control limits shown as stepped lines (solid stepped lines are 99.9% probability limits and dotted stepped lines are 95% probability limits).

**Figure 4:**
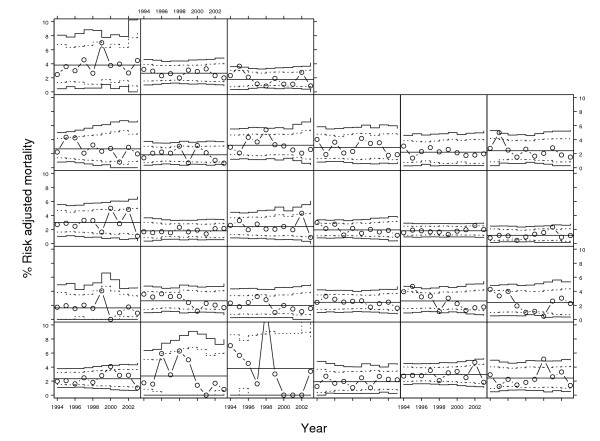
Longitudinal P-charts of risk-adjusted mortality after coronary artery bypass grafting in New York hospitals 1994–2003 for 27 hospitals (each panel). Horizontal line is the mean mortality with exact upper and lower control limits shown as stepped lines (solid stepped lines are 99.9% probability limits and dotted stepped lines are 95% probability limits).

Figure 3 and Figure 4 show the longitudinal control charts for crude and risk-adjusted mortality respectively over the ten year period for all hospitals with complete data (n = 27). In the crude longitudinal control chart there were 14 signals of special cause variation compared with 16 in the risk-adjusted charts. Table 2 compares the number of special cause signals from Figure 3 and Figure 4 as a two-by-two table. The Kappa statistic is therefore 0.61 (95% confidence interval: 0.39 to 0.83) indicating good agreement between the tests. If special cause variation on risk-adjusted mortality is taken to be the reference standard, the analysis of observed mortality has a sensitivity of 0.63 (95% confidence interval: 0.39 to 0.82) as a test for special cause variation in any given year and a specificity of 0.98 (95% confidence interval: 0.96 to 0.99).

**Table 2:** Longitudinal analysis: special cause variation in Observed and Risk-Adjusted mortality rates for coronary artery bypass grafting surgery 1994 to 2003 in New York hospitals.

Observed mortality rate	Risk Adjusted mortality rate			
	
	Special cause variation (low)	Common cause variation	Special cause variation (high)	Total
Special cause variation (low)	6	2	0	8
Common cause variation	0	250	6	256
Special cause variation (high)	0	4	4	8

Total	6	256	10	272

### Longitudinal analysis of case-mix

There are 12 instances of special cause variation in the 27 longitudinal charts. Eleven of these indicate that the expected mortality is falling.

### Longitudinal analysis of numbers of cases

When all hospitals are combined, from 1994 to 2003 there was a 20% reduction in the number of cases treated.

## Discussion

Cross-sectional analysis of crude mortality rates and risk adjusted mortality rates shows moderate agreement in identification of special causes for further investigation. This suggests that when analysing pooled data across different hospitals case-mix adjustment may make a modest difference to the analysis. Longitudinal analysis of crude mortality rates and risk adjusted mortality rates shows good agreement in the identification of special causes. In particular the specificity of analysis using crude mortality rates is high, suggesting that few spurious special causes are likely to be investigated. It is important to note that our analysis is confined to New York State's reporting of mortality rates after coronary artery bypass grafting because these data are publicly available and the generalisability of our findings should be tested in other settings.

Whilst different stakeholders (public, inspectors, media, commissioners etc) may use outcome data for different purposes [15], we believe that the ultimate purpose of outcome data is to support continual quality improvement. Improvement, by definition, requires action but since not all action leads to improvement, it is important to have guidance from theory. Shewhart's theory of variation postulates that outcomes which are consistent with common cause variation are best addressed by fundamental changes to the process of care, whereas outcomes which are consistent with special cause variation require detective work to find the cause and then action to address that cause. Outcome monitoring should support these activities with the ultimate aim of improving quality of care. Where outcome (case-mix adjusted) monitoring is used to support judgements about quality of care, often with rewards and punishments, there are serious risks of negative consequences such as distortion of data (gaming) and/or distortion of processes of care [15].

Repeated testing with a test that has a high specificity and a low sensitivity means that true positives (special cause variation) will eventually be found but few false positives will be found. Monitoring is an example of repeated testing over time. It is also important to recognise that in the context of quality improvement finding a single instance of good practice or bad practice (special cause variation) is sufficient for an organisation to learn and hence to improve. It is not necessary to find every instance of good practice or bad practice (special cause variation). High sensitivity is therefore not essential to monitoring for quality improvement. On the other hand investigation of false positives involves extensive managerial time and should be avoided, therefore high specificity is important in monitoring for quality improvement.

It follows from this that longitudinal analysis using observed (crude), mortality rates may be sufficient to guide further investigation for internal quality control. This is important because calculation of case-mix adjustment factors requires pooling of data from a number of hospitals which has inherent time delays. Hospitals may be able to conduct useful internal mortality analysis without external input.

When coupled with the observation that there was, in general, a substantial reduction in the number of cases per hospital and given that the expected mortality rate has generally fallen, it would suggest that there is some evidence of a tendency for hospitals to select lower risk patients [26]. The first report on mortality after cardiac surgery was published in November 1996[21]. Special cause variation in case-mix generally did not correspond to special cause variation in observed mortality. On the one occasion when it did, the hospital also showed special cause variation after risk-adjustment, indicating that the case-mix adjustment does not encompass the likely cause of the variation. The overall impression is that there has been a change in case-mix over time across the state, although it is hard to interpret this change in case-mix, since the multivariate logistic regression equation is recalculated each year risk factors and risk factor coefficients change from one year to the next. For example female gender is considered a risk factor in 1995 to 2002 (Odds Ratio: 1.426 to 2.097) but not in 1994 or 2003; diabetes is a risk factor in 1994, 1996, 1998 and 1999 (Odds Ratio: 1.434 to 1.726) but not in 1995, 1997 or from 2000 to 2003.

If referrals are essentially a random sample from a stable catchment population we would expect variation in case-mix to be approximately random from year to year. However coronary artery bypass surgery is an elective procedure. Both patients and their referring physicians therefore have the opportunity to choose their hospital for surgery. Surgeons may also be able to exercise choice about the kinds of patients they prefer to treat. Where elective procedures are concerned it seems likely that from year to year within a single hospital there is potential for non-random variation in case-mix. Despite this, case-mix rarely changes from one year to the next. When it does change, case-mix rarely has a great influence on hospital mortality rates. In comparison, urgent hospital admissions such as those for stroke, myocardial infarction, or surgery for fractured neck of femur are likely to be unaffected by patient or physician choice. In the absence of socio-demographic changes in the catchment area, admission patterns will represent a random sample of the at-risk population and case-mix is therefore likely to be stable. If anything, case-mix adjustment from year to year should be less important in analysis of mortality from urgent procedures. Indeed, if changes in case-mix are the explanation for special cause variation it may be important for a hospital to identify and act on this information. It follows that variation in observed mortality may be just as important an outcome to measure. Furthermore, the process of adjustment for case-mix takes account of some reasons for variation, but many other important factors (data error, inputs, processes and care pathways) are unmeasured [15]. Even if they were measured there are limits to the data that can be included in a multivariable analysis. Ultimately it is not possible to remove the effects of bias through adjustment and methods of adjustment based on logistic regression may increase bias [27].

## Conclusion

There is moderate-good agreement between signals of special cause variation between observed and risk-adjusted mortality. Analysis of observed hospital specific CABG mortality over time and with other hospitals appears to be useful in identifying special causes of variation. Case-mix adjustment may not be essential for longitudinal monitoring of outcomes using control charts.

## Competing interests

The author(s) declare that they have no competing interests.

## Authors' contributions

Tom Marshall conceived the idea and obtained the data, carried out the analysis and wrote the paper.

Mohammed A Mohammed co-authored the paper, developed the idea and undertook additional analysis.

Both authors read and approved the final manuscript.

## Pre-publication history

The pre-publication history for this paper can be accessed here:


